# SARS-CoV-2 and Other Coronaviruses in Rats, Berlin, Germany, 2023

**DOI:** 10.3201/eid3010.241079

**Published:** 2024-10

**Authors:** Kerstin Wernike, Calvin Mehl, Andrea Aebischer, Lorenz Ulrich, Mario Heising, Rainer G. Ulrich, Martin Beer

**Affiliations:** Friedrich-Loeffler-Institut, Greifswald-Insel Riems, Germany (K. Wernike, C. Mehl, A. Aebischer, L. Ulrich, R.G. Ulrich, M. Beer);; Deutsches Zentrum für Infektionsforschung, Hamburg-Lübeck-Borstel-Riems, Germany (C. Mehl, R.G. Ulrich);; SchaDe Umwelthygiene und Schädlingsbekämpfung GmbH, Berlin, Germany (M. Heising)

**Keywords:** SARS-CoV-2, severe acute respiratory syndrome coronavirus 2, coronavirus, rodents, rat, reservoir, serology, PCR, molecular typing, phylogeny, COVID-19, respiratory infections, SARS, coronavirus disease, zoonoses, viruses, Germany

## Abstract

We tested 130 rats captured in Berlin for coronaviruses. SARS-CoV-2 antibodies were detected in 1 rat, but all animals were negative by reverse transcription PCR, suggesting SARS-CoV-2 was not circulating in the rat population. However, alphacoronaviruses were found. Monitoring rodent populations helps to determine coronavirus occurrence, transmission, and zoonotic potential.

SARS-CoV-2 was initially reported in 2019 in China and spread rapidly worldwide, causing the COVID-19 pandemic in humans. Since the pandemic unfolded, the role of animals as amplifying or reservoir hosts has been hypothesized. Because of the long-term association between rodents and coronaviruses (*1*), the wide range of coronaviruses occurring in wild rodents (*2*), and the ubiquitous distribution of commensal rodents, investigations of SARS-CoV-2 and other coronaviruses in rats is warranted. In experiments that used high infection doses, rats have been reported as receptive SARS-CoV-2 hosts, particularly for the Delta variant of concern (VOC); however, experimental infections with Alpha, Beta, and Omicron variants have also been described in rats (*3*,*4*), suggesting a theoretical risk for effective transmission chains in nature. Accordingly, field studies were initiated early during the pandemic to investigate SARS-CoV-2 infections in wild rats. Indeed, serologic and molecular evidence of SARS-CoV-2 infection was found in a few animals in some studies (*2*,*3*,*5*), whereas other studies consistently reported negative results (*6*,*7*). However, all of those studies were conducted before the emergence and worldwide large-scale spread of the Omicron VOC and its subvariants. In laboratory settings, lungs from Omicron virus–infected rats showed significantly lower infectious virus titers compared with rats infected with the Delta variant (*3*), but field studies on wild rats after Omicron VOC emergence and dominance within the human populations are missing. Therefore, we investigated SARS-CoV-2 infections in Norway rats (*Rattus norvegicus*) captured in Berlin, the very densely populated (>4,000 inhabitants/km^2^) capital of Germany, during 2023, when Omicron was the dominant SARS-CoV-2 variant in the human population. Rat samples were collected during rodent pest control activities; sample collection did not require a specific permit.

We collected samples of lung and chest cavity lavage fluid from 130 Norway rats caught at 44 trapping sites (Figure 1) by rinsing the chest cavity with 1 mL phosphate-buffered saline during necropsy. We tested lavage fluids for antibodies against SARS-CoV-2 by using a receptor-binding domain (RBD)–based multispecies ELISA and a cutoff value of ≥0.3 for positivity, as previously described (*8*). We used 2 RBD protein orthologs in parallel, the wild-type virus RBD and that of the Omicron XBB1.5 variant. We prediluted the samples 1:10 as described for rodent lavage samples (*6*). 

**Figure 1:**
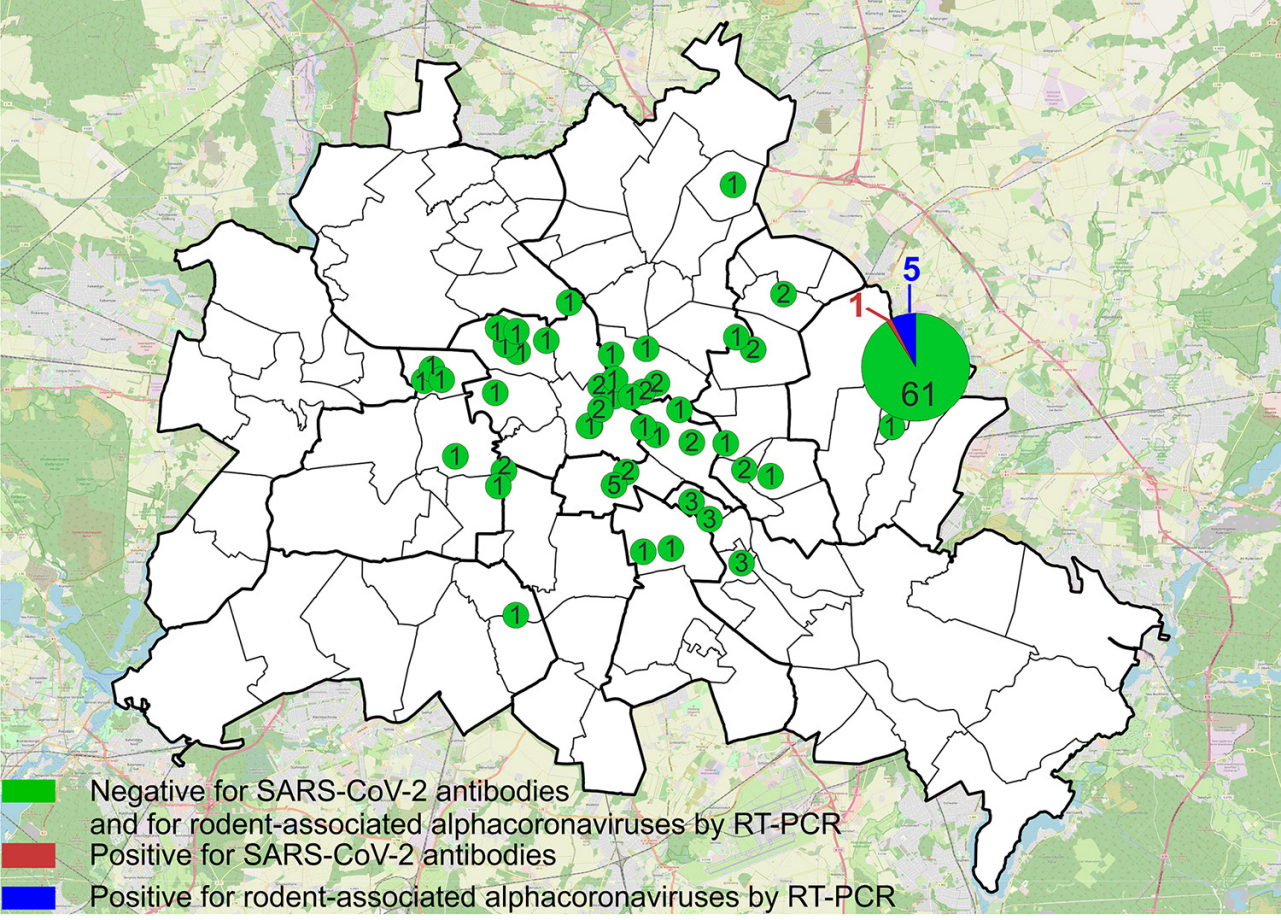
Locations of trapped rats tested in study of SARS-CoV-2 and other coronaviruses in rats, Berlin, Germany, 2023. Numbers indicate numbers of rats tested in each location. Overlay map of Berlin, in which the circles were printed, was retrieved from Geoportal Berlin, dataset Geoportal Berlin/Ortsteile von Berlin (https://daten.odis-berlin.de/de/dataset/ortsteile), data license Germany–attribution–Version 2.0 (https://www.govdata.de/dl-de/by-2-0). Map of the area surrounding Berlin was created by using OpenStreetMap (https://www.openstreetmap.org). RT-PCR, reverse transcription PCR.

One of 130 rats tested positive; the optical density values were 1.16 (wild-type RBD) and 1.53 (Omicron RBD) (Appendix Figure). To confirm the positive result, we tested the sample by using a surrogate virus neutralization test (cPass SARS-CoV-2 Neutralization Antibody Detection Kit; GenScript, https://www.genscript.com) and 2 different RBD orthologs according to the manufacturer’s instructions (cutoff for positivity was >30% inhibition). That test, in its original composition, enables the detection of antibodies against wild-type SARS-CoV-2 and all VOCs except Omicron. For Omicron and its subvariants, we used a specific RBD provided by the manufacturer (GeneScript). The ELISA-positive rat sample was positive against Omicron-specific RBD in the neutralization test (33.9% inhibition for Omicron, 23.4% for wild-type RBD), suggesting the rat had a previous infection with an Omicron subvariant. However, only 1 rat tested positive, indicating a single spillover event from humans into the rat population and lack of autonomous virus circulation in rats, especially considering 66 additional rats were caught in the same building as the seroreactive animal (Figure 1), and all of those tested negative. Potential cross-reactivity with other coronaviruses could account for the single positive result, although cross-reactivity of some animal coronaviruses was excluded during initial validation of the RBD-based ELISA (*8*).

To further confirm that no virus circulated in the sampled rat population, we tested lung samples by using SARS-CoV-2–specific real-time reverse transcription PCR (RT-PCR) targeting the RNA-dependent RNA polymerase (*RdRp*) gene (*9*) and by using an *RdRp*-based, generic pancoronavirus RT-PCR (*10*). Using the SARS-CoV-2–specific test, all samples were negative, verifying the absence of SARS-CoV-2. Nevertheless, 5 lung samples were positive in the pancoronavirus RT-PCR; all 5 animals were trapped at the same location (Figure 1). For further characterization, we sequenced the RT-PCR products in both directions by using the PCR amplification primers. We deposited the sequences in GenBank (accession nos. OR854629–33) and compared them with other representative coronavirus sequences obtained from GenBank. Virus typing according to the partial *RdRp* sequences revealed that the viruses found in Berlin rats belonged to the genus *Alphacoronavirus* and were closely related to each other (99.4%–100.0% nucleotide sequence identity) and to the Lucheng Rn rat coronavirus (Figure 2). Hence, in contrast to SARS-CoV-2, rodent-associated alphacoronaviruses were circulating within the Berlin rat population, which agrees with previous studies of coronaviruses in rats in other locations (*2*,*5*).

**Figure 2:**
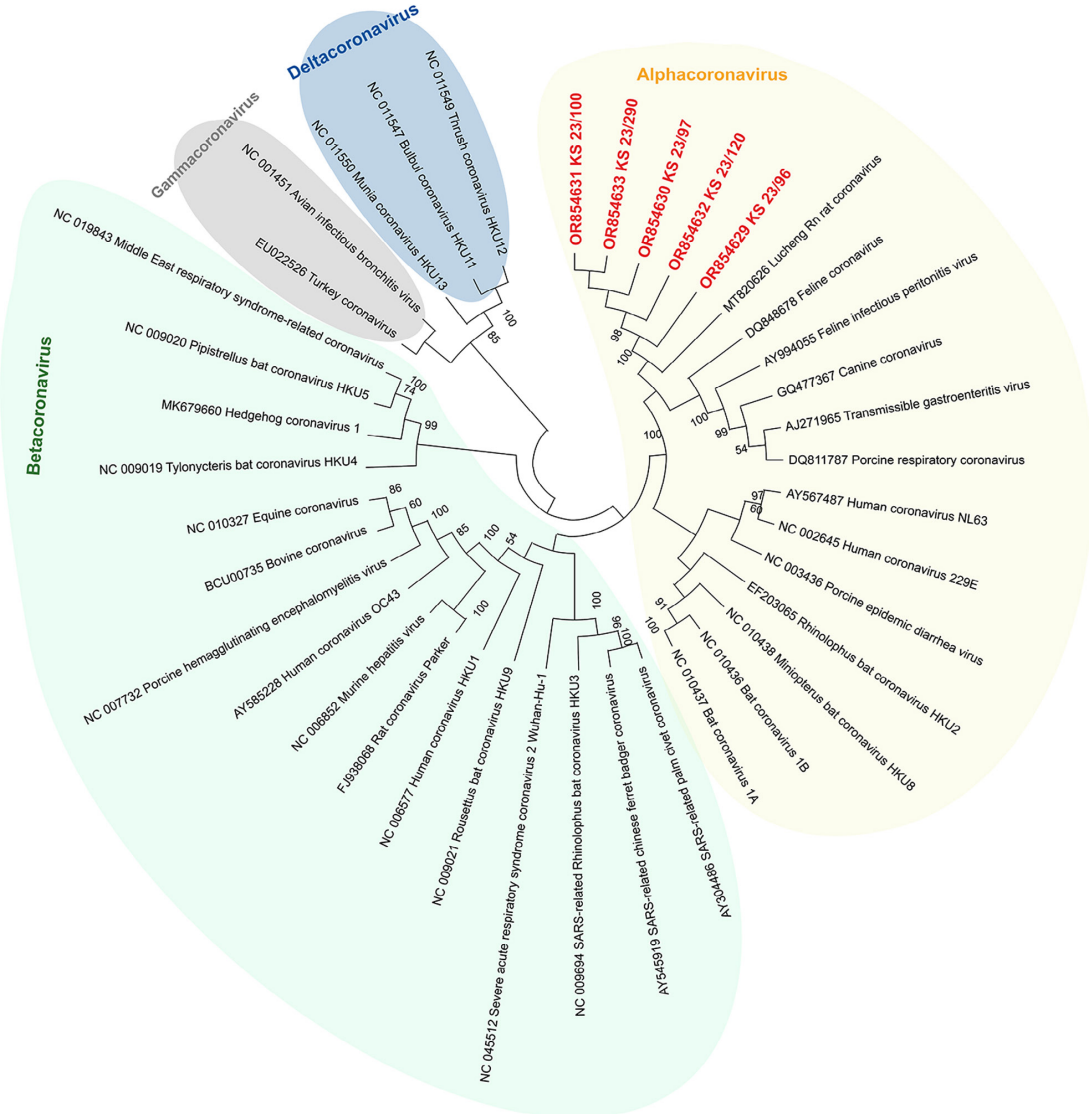
Phylogenetic analysis of SARS-CoV-2 and other coronaviruses in rats, Berlin, Germany, 2023. Partial sequences of the RNA-dependent RNA polymerase gene from coronaviruses isolated from rats in Berlin (red text) were compared with other coronavirus sequences obtained from GenBank. Background colors indicate viruses belonging to the same coronavirus genus. The maximum-likelihood tree was calculated by using MEGA X software (https://www.megasoftware.net). Statistical support for nodes was obtained by bootstrapping (1,000 replicates); only bootstrap values >50% are shown. GenBank accession numbers are provided. Tree not drawn to scale.

In conclusion, research into rodent coronaviruses contributes to a broader understanding of those viruses and aids in the development of strategies for managing both animal and public health. Coronavirus monitoring of rodent populations aids in determining virus occurrence, transmission characteristics, pathogenesis, and zoonotic potential.

Appendix Additional information for SARS-CoV-2 and other coronaviruses in rats, Berlin, Germany, 2023.

## References

[1] Tsoleridis T, Chappell JG, Onianwa O, Marston DA, Fooks AR, Monchatre-Leroy E, et al. Shared common ancestry of rodent alphacoronaviruses sampled globally. Viruses. 2019;11:125. 10.3390/v11020125 30704076 PMC6409636

[2] Fisher AM, Airey G, Liu Y, Gemmell M, Thomas J, Bentley EG, et al. The ecology of viruses in urban rodents with a focus on SARS-CoV-2. Emerg Microbes Infect. 2023;12:2217940. 10.1080/22221751.2023.2217940 37219409 PMC10262798

[3] Wang Y, Lenoch J, Kohler D, DeLiberto TJ, Tang CY, Li T, et al. SARS-CoV-2 exposure in Norway rats (*Rattus norvegicus*) from New York City. MBio. 2023;14:e0362122. 10.1128/mbio.03621-22 36892291 PMC10127689

[4] Zhang C, Cui H, Li E, Guo Z, Wang T, Yan F, et al. The SARS-CoV-2 B.1.351 variant can transmit in rats but not in mice. Front Immunol. 2022;13:869809. 10.3389/fimmu.2022.869809 35572504 PMC9095975

[5] Miot EF, Worthington BM, Ng KH, de Lataillade LG, Pierce MP, Liao Y, et al. Surveillance of rodent pests for SARS-CoV-2 and other coronaviruses, Hong Kong. Emerg Infect Dis. 2022;28:467–70. 10.3201/eid2802.211586 35076003 PMC8798707

[6] Wernike K, Drewes S, Mehl C, Hesse C, Imholt C, Jacob J, et al. No evidence for the presence of SARS-CoV-2 in bank voles and other rodents in Germany, 2020–2022. Pathogens. 2022;11:1112. 10.3390/pathogens11101112 36297169 PMC9610409

[7] Colombo VC, Sluydts V, Mariën J, Vanden Broecke B, Van Houtte N, Leirs W, et al. SARS-CoV-2 surveillance in Norway rats (*Rattus norvegicus*) from Antwerp sewer system, Belgium. Transbound Emerg Dis. 2022;69:3016–21. 10.1111/tbed.14219 34224205 PMC8447303

[8] Wernike K, Aebischer A, Michelitsch A, Hoffmann D, Freuling C, Balkema-Buschmann A, et al. Multi-species ELISA for the detection of antibodies against SARS-CoV-2 in animals. Transbound Emerg Dis. 2021;68:1779–85. 10.1111/tbed.13926 33191578 PMC7753575

[9] World Health Organization. Real-time RT-PCR assays for the detection of SARS-CoV-2 [cited 2024 Sep 1]. https://www.who.int/docs/default-source/coronaviruse/real-time-rt-pcr-assays-for-the-detection-of-sars-cov-2-institut-pasteur-paris.pdf

[10] Chidoti V, De Nys H, Pinarello V, Mashura G, Missé D, Guerrini L, et al. Longitudinal survey of coronavirus circulation and diversity in insectivorous bat colonies in Zimbabwe. Viruses. 2022;14:781. 10.3390/v14040781 35458511 PMC9031365

