# Supplementary material for: SARS-CoV-2 and Other Coronaviruses in Rats, Berlin, Germany, 2023
Source: Emerg Infect Dis. 2024 Oct;30(10):2205–8. doi: 10.3201/eid3010.241079 (PMC11431905; doi:10.3201/eid3010.241079)
Supplement: Appendix — Additional information for SARS-CoV-2 and other coronaviruses in rats, Berlin, Germany, 2023. [file 24-1079-Techapp-s1.pdf]

Article DOI: https://doi.org/10.3201/eid3010.241079

EID cannot ensure accessibility for Supplemental Materials supplied by authors. Readers who have difficulty accessing supplementary content should contact the authors for assistance.

SARS-CoV-2 and Other Coronaviruses in Rats, Berlin, Germany, 2023

Appendix

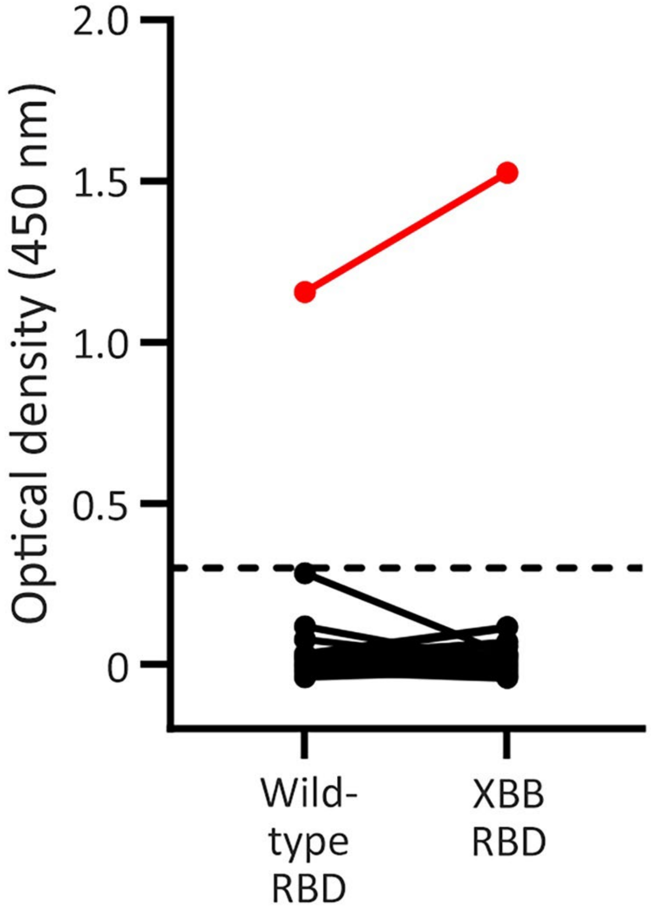

Appendix Figure. Results of the coronavirus RBD-based ELISA in study of SARS-CoV-2 and other coronaviruses in rats, Berlin, Germany, 2023. Samples of rat chest cavity lavage fluid were tested by ELISA in parallel by using the SARS-CoV-2 wild-type or Omicron XBB variant RBD orthologs, and results of individual samples are connected by lines. Red line indicates the SARS-CoV-2–seropositive rat. Dashed line indicates the cutoff value for positivity. RBD, receptor-binding domain.
